# Influence of nursing staff working hours on stress levels during the COVID-19 pandemic: A cross-sectional online survey

**DOI:** 10.1007/s16024-021-00354-y

**Published:** 2021-09-10

**Authors:** Manuela Hoedl, Silvia Bauer, Doris Eglseer

**Affiliations:** Institute of Nursing Science, Medical University of Graz, Universitaetsplatz4/3, 8010 Graz, Austria

**Keywords:** Cross-sectional studies, Nursing staff, Psychological stress, Shift work schedule, Work schedule tolerance, Querschnittstudie, Pflegepersonal, Stress, Schichtarbeit, Auswirkungen der Arbeitszeit

## Abstract

**Background:**

Working as a nurse means being able to provide high-quality care 24/7. Studies have shown that the average number of working hours per week is a significant predictor of stress and that the severity of the coronavirus disease 2019 (COVID-19) pandemic has increased the nurses’ stress levels.

**Objective:**

The aim of this study was to investigate the influence of the nursing staff’s working hours during the COVID-19 pandemic on the perceived level of stress.

**Method:**

We carried out an online cross-sectional survey and measured the stress level with the perceived stress scale.

**Results:**

Most of the nurses experienced a moderate level of stress. We identified a statistically significant association between increased numbers of working hours per week and the nurses’ perceived stress level. In addition, 15% of the nurses who had worked more than 40 h reported experiencing a high level of stress.

**Conclusion:**

These results reflect the negative consequences of prolonged working hours. For this reason, a (inter)national discussion is needed on the topic of restricting the working hours of healthcare workers during such pandemics. This discussion can improve the health and safety of healthcare workers, patients and members of the general population.

## Abstract (de)

**Hintergrund:**

Als Pflegeperson zu arbeiten bedeutet, rund um die Uhr eine qualitativ hochwertige Versorgung anbieten zu können. Studien haben gezeigt, dass die durchschnittliche Anzahl der Arbeitsstunden pro Woche ein signifikanter Prädiktor für Stress ist und dass der Schweregrad der Coronavirus-Krankheit-2019(COVID-19)-Pandemie den Stresspegel der Pflegepersonen erhöht.

**Ziel der Arbeit:**

Ziel dieser Studie war es, den Einfluss der Arbeitszeit des Pflegepersonals während der COVID-19-Pandemie auf das wahrgenommene Stressniveau zu untersuchen.

**Methode:**

Wir haben eine Online-Querschnittserhebung durchgeführt und das Stressniveau mit der Perceived Stress Scale erhoben.

**Ergebnisse:**

Die meisten Pflegepersonen gaben ein moderates Stresslevel an. Die Steigerung der Arbeitsstunden pro Woche hing statistisch signifikant mit dem wahrgenommenen Stresslevel zusammen. Darüber hinaus gaben 15 % der Pflegepersonen, die mehr als 40 h gearbeitet hatten, an, unter hohem Stress zu leiden.

**Schlussfolgerung:**

Diese Ergebnisse spiegeln die negativen Folgen einer längeren Arbeitszeit wider. Aus diesem Grund ist eine (inter)nationale Diskussion zum Thema der Einschränkung der Arbeitszeit von Beschäftigten im Gesundheitswesen während solcher Pandemien erforderlich. Diese Diskussion kann die Gesundheit und Sicherheit der Beschäftigten im Gesundheitswesen, der Patientinnen sowie der Bevölkerung verbessern.

## Introduction

The healthcare system has been organized to ensure that 24‑h per day services are provided 7 days a week. Within the healthcare system, nurses represent the largest professional group; therefore, the overall quality of the healthcare system largely depends on the nurses’ performance. Nevertheless, the ability of the nurse to provide high-quality care is strongly associated with the health of nursing staff (Rosa et al. 2018).

## Problem statement

To work as a nurse, either as a qualified nurse or nursing aide, individuals need to be responsive and highly accountable 24/7 in order to fulfil the health service requirements (Rosa et al. 2018). In the European Union, the 2003 Working Time Directive sets limits of 48 h per week, an amount of working time that includes overtime, calculated as an average over a maximum of 4 months (European Parliament and Union 2003). This directive also defines a daily amount of rest, i.e. a minimum 11 h, and a weekly amount of rest, i.e. normally 24 h plus the 11‑h daily rest period and the amount of time spent performing work at night (European Parliament and Union 2003).

However, the directive allows deviations or exemptions in Article 17 paragraph 3 (European Parliament and Union 2003); these deviation or exemptions were required during the recent coronavirus disease 2019 (COVID-19) pandemic. This might have led to prolonged/irregular working hours for nursing staff. Such prolonged/irregular working hours can include working periods that exceed the conventional working hours, overtime, shift work, night work and on-call scheduling.

These long/irregular hours worked by nurses can have negative consequences on the health and safety of both nurses and patients. Several studies have shown that such prolonged/irregular working hours can result in nurses having a lower ability to detect adverse changes in their patients or address them in a timely manner (Trinkoff et al. 2011), reduced or lacking patient safety, fair to poor quality of care offered, more care activities left undone (Griffiths et al. 2014), or adverse nursing outcomes such as complaints from patients or families (Son et al. 2019).

Another study reported several negative consequences for the nurses, such as increased emotional and mental fatigue, the disruption of normal sleeping and waking hours, depression and various illnesses such as musculoskeletal disorders (Harris et al. 2015). These disruptions could also result in a diminished capacity to manage the workload, job dissatisfaction, burnout, absenteeism as well as the recruitment and retention of nursing staff (Messenger and Vidal 2015; Yildirim and Aycan 2008). Furthermore, irregular work schedules and work overload can lead to professional and personal conflicts, which may affect personal and family responsibilities in the long run and decrease the nurses’ levels of satisfaction with their work and lives (Messenger and Vidal 2015; Yildirim and Aycan 2008).

One main consequence of long/irregular hours worked by nurses is stress. Stress can be explained as the degree to which life is experienced as unpredictable, uncontrollable and overloaded (Klein et al. 2016), of which all three aspects (unpredictable, uncontrollable as well as overloaded) can be linked to the COVID-19 pandemic. Four studies have been carried out to investigate the level of stress of nurses. Whereas Almazan et al. concluded that the average number of working hours per week was a significant predictor of stress (2019) the other study reported that high stress can lead to a decline in the quality of nursing care (Keykaleh et al. 2018). Another study reported that 70% of the healthcare workers experienced emotional distress (Riguzzi and Gashi 2021). In addition, caring for persons affected by COVID-19- or suspected cases might also influence the nurses’ level of stress (Mo et al. 2020). The four aforementioned studies were conducted in Saudi Arabia, Iran, Switzerland and China, which do not belong to the European Union, and might therefore have different working time directives than the European Union. So studies focusing on prolonged/irregular working hours in nurses during the COVID-19 pandemic within the European Union are still missing.

Overall, the consequences of nurses working prolonged/irregular working hours are enormous and also result in increased stress among nurses, which might be reinforced by caring for COVID-19 patients. Even though the European Union has a working time directive, this can be legally overridden during the COVID-19 pandemic in order to ensure the continuity of the healthcare service.

## Aims

Therefore, we investigated (1) if a change had occurred in the nurses’ working hours during the COVID-19 pandemic as compared to the hours listed in their employment contract and (2) the influence of the nursing staff’s working hours during COVID-19 pandemic on the perceived level of stress.

## Material and methods

### Design

For this cross-sectional study, we used an online questionnaire, which was distributed using a snowball sampling method and with the aid of social media. The online survey was opened on 12 May 2020 and closed on 13 July 2020.

### Setting and sample

The authors invited Austrian nursing staff, including nurses, nursing aids and care aids who were working at the bedside with patients/residents during the COVID-19 pandemic. We invited nursing staff working in different settings, such as hospital and long-term care facilities. Nursing leaders, managers, or directors were not invited to take part in the survey, so that we could obtain detailed insight into nursing practice during this pandemic.

### Data collection instrument

We collected demographic sample characteristics such as the year of birth and gender. In addition, we asked the nurses how many hours they were employed per week and to estimate the average number of hours they had worked per week since the outbreak of the COVID-19 pandemic in Austria (i.e. mid-March 2020). Possible answers to this question were: < 10 h, 10–20 h, 21–30 h, 31–40 h, or > 40 h.

On a structural level, we collected data on the perceived change in the working hours due to the COVID-19 pandemic (possible answers: yes, working hours were prolonged; yes, working hours were reduced; yes, working hours were changed; no change).

To assess the perceived stress level among the nurses, we used the German version of the perceived stress scale (PSS) on the outcome level (Cohen et al. 1983; Schneider et al. 2017). The PSS has good psychometric properties (Cohen et al. 1983) and consists of ten items, which highlights its practicability (Klein et al. 2016). Each item can be rated on a 5-point Likert scale (0 = never; 1 = almost never; 2 = sometimes; 3 = fairly often; 4 = very often) (Klein et al. 2016).

Therefore, PSS scores can range from 0 to 40, with higher scores indicating higher perceived stress. In addition, categories can be constructed by using the sum score with scores of 0–13 to indicate a low perceived stress level, 14–26 moderate perceived stress level and 27–40 high perceived level of stress (Cohen et al. 1983; Schneider et al. 2017).

The PSS is a frequently used instrument for assessing stress in nursing staff (Aalaa et al. 2017; Cicolini et al. 2016; dos Santos et al. 2016; Montanari et al. 2019; Peters et al. 2020; Rayan et al. 2019; Uzun and Sevinc 2015; Wright 2018).

### Data analysis

Data analysis was conducted by using SPSS 26 (IBM Corp (IBM Corp. Released 2019, Armonk, NY, USA). Released 2019). Three participants were excluded from this analysis because they were 69–70 years of age, which is older than the retirement age in Austria.

We calculated descriptive statistics, including percentages for categorical variables and means and a standard deviation for metric variables. The χ^2^-test was used to assess categorical variables and, depending on the number of answer categories, Cramer (V) or the contingency coefficient (CC) were reported as a measure of the effect size. In order to validate our findings with respect to the influence of changes in the working hours on the PSS sum, we conducted an independent samples Kruskal-Wallis test. We set the *p*-values < 0.05 as statistically significant.

### Ethics

The ethics committee of a university approved the study (32-386 ex 19/20). All participants had to click on “Yes”, if they wanted to participate in the survey. The data collection was anonymous, and the collected data were stored on the university server.

## Results

### Sample characteristics

A total of 2602 nurses participated in the survey, most of whom were employed between 31 and 40 h per week (Table 1). Of this number, 75.6% of the nurses reported changes in their working hours during the COVID-19 pandemic. Nearly 60% of the nurses described changes, e.g. due to reorganization, and 13.3% reported that their working hours were prolonged during the COVID-19 pandemic. Of these nurses two thirds were caring for a suspected case or a person infected with COVID-19 during their working hours.

**Table 1:** Sample characteristics

Sample characteristics	Nursing staff (*N* = 2602)
*Female %*	83.7
*Mean age in years (SD)*	38 (11)
*Number of hours per week employed %*	
< 10 h	3.3
10–20 h	8.8
21–30 h	16.6
31–40 h	71.4
*Change in working hours %*	
Yes, prolonged	13.3
Yes, reduced	4.1
Yes, changed, e.g. through reorganization	58.3
No changes	24.4
*Caring for a suspected case or a person infected with COVID-19%*	
Yes	66.5
No	33.5

### Comparison of hours of employment and current working hours

In order to give a detailed insight, the hours of employment per week were compared to the average number of working hours per week during the COVID-19 pandemic (Table 2).

**Table 2:** Comparison of hours of employment per week and average number of working hours per week during the COVID-19 pandemic

Hours of employment per week*	Average working hours per week during COVID-19 pandemic %				
	< 10	10–20	21–30	31–40	> 40
< 10 (*n* = 85)	30.6	3.5	8.2	20.0	37.6
10–20 (*n* = 228)	5.3	64.0	19.3	5.3	6.1
21–30 (*n* = 431)	1.6	13.5	59.2	20.2	5.6
31–40 (*n* = 1858)	2.3	2.3	8.8	53.7	33.0
Total (*N* = 2602)	3.3	9.6	18.1	42.8	26.2

* *p* < 0.05

We identified a statistically significant association between the hours of employment and the average number of working hours during the COVID-19 pandemic among nursing staff (*V* = 0.487, *p* = 0.000). About two thirds of the nurses who were employed less than 10 h worked more than their hours of employment. Most of the nurses who were employed for more than 10 h worked the hours for which they were employed. One third of the nurses that were employed 31–40 h worked more than 40 h per week during the COVID-19 pandemic.

### Influence of working hours on stress

The majority of nurses experienced a moderate level of stress (Table 3). We identified a statistically significant association between an increase in the number of working hours per week and the perceived stress level of the nurses (*V* = 0.078, *p* = 0.000). About 15% of the nurses who worked more than 40 h per week reported experiencing a high level of stress.

**Table 3:** Association between the average number of working hours per week during the COVID-19 pandemic and perceived stress (PSS) among nursing staff

Average working hours per week during COVID-19*	PSS categories %		
	Low	Moderate	High
< 10 (*n* = 87)	35.6	58.6	5.7
10–20 (*n* = 249)	39.4	52.6	8.0
21–30 (*n* = 470)	34.7	57.2	8.1
31–40 (*n* = 1113)	32.5	58.1	9.3
> 40 (*n* = 683)	29.0	55.6	15.4
Total (*N* = 2602)	32.7	56.8	10.5

*PSS *perceived stress scale

* *p* < 0.05

In order to give a detailed insight into how the change in working hours influenced stress, Table 4 shows the influence of the change in working hours during the COVID-19 pandemic on the nurses’ stress levels. We defined the working hours as changed when the hours of employment per week and average working hours per week during COVID-19 pandemic differed. Some nurses might have worked less, the same, or more during the COVID-19 pandemic than their official employment contract stipulated.

**Table 4:** Association between changed working hours during the COVID-19 pandemic and perceived level of stress (PSS) among nursing staff

Change in working hours*	PSS categories %		
	Low	Moderate	High
Reduced (*n* = 325)	37.5	53.5	8.9
Same (*n* = 1424)	34.6	56.7	8.8
Increased (*n* = 853)	27.9	58.3	13.8

* *p* < 0.05

No matter how the working hours had changed, more than half of the nurses experienced moderate stress (53.5–58.3%). We found a statistically significant association between the three groups with respect to the perceived stress level (CC = 0.097, *p* = 0.000), meaning that nurses with increased working hours experienced higher stress than nurses with reduced or the same working hours.

In order to validate these findings, we calculated an independent sample Kruskal-Wallis test to assess the effect of the change in the working hours on the PSS sum score. We found a statistically significant increase in nursing staff stress level when the hours increased as compared to when the hours were reduced (*p* = 0.001) or stayed the same (*p* = 0.000). We found no statistically significant difference in the stress level when we compared nurses who worked reduced or the same working hours (*p* = 0.850).

## Discussion

This study was carried out to investigate the influence of nursing staff working hours during the COVID-19 pandemic on the perceived level of stress. Three quarters of the nurses reported changes in their working hours during COVID-19. We identified a statistically significant association between the hours of employment and the average working hours during the COVID-19 pandemic among nursing staff. About two thirds of the nurses who were employed less than 10 h worked more than their hours of employment, and one third of the nurses who were employed 31–40 h worked more than 40 h during the COVID-19 pandemic. The majority of nurses experienced a moderate level of stress. We identified a statistically significant association between an increase in working hours per week and the nurses’ perceived stress levels. In addition, 15% of the nurses who worked more than 40 h reported experiencing a high level of stress. We also found that nurses with increased working hours experienced higher stress than nurses with reduced or the same working hours.

Our results show that most nursing staff work between 31–40 h per week. This could be explained by the high number of younger nurses who participated in the study, and who may not yet have family responsibilities. This finding is underpinned by a study which highlighted that 39% of the nursing staff in Austria are younger than 40 years of age (Federal Ministry for Social Affairs Health Care and Consumer Protection 2019).

When the actual working hours were compared to the hours of employment, we saw that between 25.7% in the group that were employed 21–30 hours up to 37.3% in the group that were emplyoed 10 or less hours worked for more hours than they were legally obliged to. This is surprising, as the Austrian government recommended in mid-March that all unnecessary health treatments, e.g. knee replacement surgery, should be postponed, which should have decreased the number of hospital patients (Federal Ministry of Social Affairs, Health, Care and Consumer Protection, *Bundesministerium für Soziales Gesundheit Pflege und Konsumentenschutz* 2020); however, a more recent study on COVID-19 among healthcare workers reported that nearly one third stated that they have to work more than usual (Riguzzi and Gashi 2021).

The majority of nurses in our study experienced a moderate level of stress. This is in line with two studies, which were conducted before COVID-19 pandemic and reported a moderate stress level among nurses (Almazan et al. 2019; Keykaleh et al. 2018). In a more recent study 70% of the participants experienced emotional distress (Riguzzi and Gashi 2021). In this study they used one question on how worried the participants felt, because of the possibility of (1) “getting COVID-19 yourself,” (2) “family/friends getting COVID-19,” and (3) “numerous cases of death among old and sick people due to COVID-19”, which can be rated by a 4-point Likert scale. In addition, emotional distress can be seen as one aspect of stress beside physical or mental stress etc. In our study as well as in the study from Almazan et al. the PSS was used and Keykaleh et al. used the nurse’s job stress questionnaire, which can both be seen as general stress measurement instruments. This might explain the different results.

We could also show that about 15% of the nurses who worked more than 40 h reported a high perceived level of stress. These findings are in line with those of previous studies not focused on COVID-19 that linked long/irregular working hours to emotional and mental fatigue, disruptions of normal sleeping and waking hours, depression and various illnesses, such as musculoskeletal disorders (Harris et al. 2015). In addition, the health and safety of the patients decreased as the nurses’ working hours increase (Griffiths et al. 2014; Son et al. 2019; Trinkoff et al. 2011); however, a more detailed insight, in e.g. specific wards such as intensive care units (ICU), for which a higher stress level can be assumed, was not possible with our data. Even though this would have enhanced the interpretability of our results.

This is an especially interesting result in times of a pandemic. If patient safety indicators such as accidents, near misses, failures, or errors in healthcare occur during a pandemic, this might result in more severe consequences than in normal times. In addition, such failures to ensure patient safety could actually increase the effect of the pandemic on the general population, through healthcare workers who (unintentionally) act as coronavirus superspreaders. In any case, these results indicate that a societal and political discussion about working hour regulations for healthcare workers during such a pandemic should be initiated, as these might increase the health and safety of the healthcare workers, the patients and members of the general population.

## Limitations

We assessed the nurses’ working hours during the COVID-19 pandemic, which might not reflect the nurses’ workload during normal working hours. Studies have shown that the more patients a nurse has to care for, the higher the intensity of work, which can result in an increased risk of accidents and work-related stress, high levels of sick leave and absenteeism (Messenger and Vidal 2015) and, consequently, injuries and diseases including fatigue and burnout. On the other hand, a recent study concluded that the average number of working hours per week was a significant predictor of stress (Almazan et al. 2019). Another limitation of this study is that we did not assess other factors, such as the working climate, which might also have an influence on the perceived level of stress and, specifically, in this kind of pandemic. One more limitation is that we used a convenience sample. Therefore, our results cannot be generalized to the entire country of Austria. Moreover, this might have led to an underestimation of the effects, as highly stressed nurses might not be willing or do not have the energy to participate. Furthermore, older nursing staff might not have the same access to online surveys than younger nursing staff; however, this initial study conducted during a pandemic provides us with important insights into this topic that encourage us and other researchers to repeat the study with larger sample sizes.

## Conclusion

This study was carried out to investigate the influence of nursing staff working hours during the COVID-19 pandemic on the perceived level of stress. We found a statistically significant association between an increase in working hours and the level of perceived stress among nursing staff.

We believe that these results reflect the negative consequences of prolonged working hours. For this reason, a (inter)national discussion is needed on the topic of restricting the working hours of healthcare workers during such pandemics. This discussion can improve the health and safety of the healthcare workers, the patients, as well as members of the general population and can even save lives.

### Implications for clinical practice

As we have shown that prolonged working hours are associated with a growing stress level among nurses, during such pandemics, the awareness with respect to more and prolonged breaks should be increased. This should be one aim in and for clinical practice for the respective nursing works council. Moreover, if possible temporary workers should be employed instead of increasing nursing staff working hours.
